# Supplementary material for: An Application of the Theory of Planned Behaviour to Predict Intention to Consume Plant-Based Yogurt Alternatives
Source: Foods. 2021 Jan 12;10(1):148. doi: 10.3390/foods10010148 (PMC7828233; doi:10.3390/foods10010148)
Supplement: Supplementary file 1 [file foods-10-00148-s001.pdf]

Supplement Material

Table 1. Proposed theoretical model constructs and measuring items with their source of adoption.

Constructs	Measuring Item	Source of Adoption
**Attitude**		[39,43]
ATT1	I think consuming plant-based yogurt alternatives is a sustainable choice.	
ATT2	I think that consuming plant-based yogurt alternatives is environmentally friendly.	
ATT3	I think that plant-based yogurt alternatives have digestive benefits.	
ATT4	I think that plant-based yogurt alternatives are beneficial for my health.	
ATT5	I think consuming plant-based yogurt alternatives is better for animal welfare.	
ATT6	Consuming plant-based yogurt alternatives makes me feel good.	
**Subjective norms**		[25,44]
SN1	My friends think I should consume plant-based yogurt alternatives.	
SN2	My family thinks I should consume plant-based yogurt alternatives.	
SN3	My colleagues/classmates think I should consume plant-based yogurt alternatives.	
**Objective knowledge of plant-based yogurt alternatives**		[34,45]
KN1	Plant-based yogurt alternatives are exclusively composed of plant-originated products.	
KN2	Plant-based yogurt alternatives are fermented.	
KN3	Plant-based yogurt alternatives contains probiotics.	
KN4	Plant-based yogurt alternatives have nutritional values similar to dairy yogurt.	
**Perceived behavioural control (self-efficacy)**		[44]
PBC1	It's up to me whether I eat plant-based yogurt alternative products or not.	
PBC2	I am confident that, if I want to, I can easily consume a plant-based yogurt alternative.	
**Perceived barriers**		[39]
PB1	Plant-based yogurt alternatives are too expensive.	
PB2	Plant-based yogurt alternatives are not easily available in my regular grocery stores.	
PB3	Plant-based yogurt alternatives are not good for my health.	
PB4	Information about plant-based yogurt alternatives is difficult to access.	
PB5	Plant-based yogurt alternatives are not tasty.	
PB6	I don't like to try new foods.	
**Perceived sensory attributes**		[46]
PSA1	I like the smell of plant-based yogurt alternative products.	
PSA2	I like the taste of plant-based yogurt alternative products.	
PSA3	I like the mouthfeel of plant-based yogurt alternative products.	
PSA4	I like the texture of plant-based yogurt alternative products.	
**Intention**		[44]
Int1	The likelihood that I eat plant-based yogurt alternative products in the next two weeks is high.	
Int2	I am planning to eat plant-based yogurt alternative products in the next two weeks.	
**Behaviour**		[30]
B	How often do you consume plant-based yogurt alternatives?	

Table 2. Descriptive analysis and results of exploratory factor analysis (factor loading, reliability, volatility and validity).

Constructs	Items	Median	IQR	Factor loading	Cronbach's α	AVE*	CR
Attitude	ATT1	4	1	0.810	0.827	0.617	0.865
	ATT2	4	1	0.789			
	ATT3	4	1	0.711			
	ATT4	4	1	0.827			
Subjective norms	SN1	3	1	0.886	0.861	0.766	0.907
	SN2	3	1	0.855			
	SN3	3	1	0.885			
Perceived behavioural control (self-efficacy)	PBC1	5	1	0.800	0.720	0.582	0.735
	PBC2	5	1	0.724			
Objective knowledge	KN	2	3	1.00 (fixed)	-	-	-
Perceived barriers (reversed)	PB1	2	1	0.719	0.745	0.559	0.835
	PB2	2	2	0.783			
	PB3	3	2	0.714			
	PB4	2	2	0.773			
Perceived sensory attributes	PSA1	5	1	0.710	0.879	0.653	0.882
	PSA2	4	2	0.781			
	PSA3	4	1	0.862			
	PSA4	4	1	0.869			
Intention	INT1	4	1	0.840	0.939	0.716	0.835
	INT2	4	2	0.853			
Behaviour	B	3	3	1.00 (fixed)	-	-	-

Note: AVE = Average Variance Extracted, AVE* calculated as ∑ SMC/ (∑ SMC + ∑standard measurement error); CR = Composite Reliability; IQR = Interquartile range.

Variables with factor loading < 0.70 were not assigned: attitudes – animal welfare (ATT5) and makes me feel good (ATT6); perceived barrier – tasty (PB5) and new foods (PB6).

Behaviour was measured by the following item: 'How often do you consume plant-based yogurt alternative products?': 1 = Less than once a month; 2 = Once a month; 3 = Once every 2 to 3 weeks; 4 = Once a week; 5 = Once every 2 to 3 days; and 6 = Every day. .

Attitude, subjective norms, perceived behavioural control (self-efficacy), perceived barrier, perceived sensory attributes and intention was measured on a 5-point Likert scale with 'Strongly disagree', 'Disagree', 'Neither disagree nor agree', 'Agree', 'Strongly Agree'.

Objective knowledge of plant-based yogurt alternatives was measured with 'True', 'False' and 'I don't know' and later coded as 1 for correct and 0 as incorrect and computed as the total number of correct responses, thus ranging from 0 to 4.

Table 3. Correlation among the constructs.

Constructs	KN	A	SN	PBC	PB	PSA	INT	B
Objective Knowledge (KN)	**1**							
Attitude (A)	0.074	**0.785**						
Subjective norms (SN)	0.041	0.130*	**0.875**					
Perceived behavioural control (self-efficacy) (PBC)	0.011	0.153*	0.017	**0.762**				
Perceived barriers (PB)	0.083	0.010	-0.029	0.035	**0.747**			
Perceived sensory attributes (PSA)	0.127*	0.272**	0.260**	0.366**	0.061	**0.808**		
Intention (INT)	0.034	0.298**	0.228**	0.336**	0.007	0.445**	**0.846**	
Behaviour (B)	0.035	0.321***	0.140*	0.252***	0.51	0.294***	0.662***	**1**

Note: *significant effect at $p < 0.05$, **significant effect at $p < 0.01$, ***significant effect at $p < 0.001$ (Two-tailed). Bold values represent the square root of AVE; correlation were estimated through Pearson's Correlation test. .

Table 4. The goodness of fit indices.

Fit Indices	TPB Framework	Proposed Theoretical Framework	Norm [56]
χ^2	185.825	339.659	N/A
Scaled χ^2/df	3.954	1.963	>1 and <5
GFI	0.888	0.898	≥0.90
TLI	0.880	0.924	≥0.90
CFI	0.915	0.937	≥0.90
RMSEA	0.106	0.060	≤0.80
Adjusted R^2 (INT)	0.255	0.292	
Adjusted R^2 (B)	0.473	0.459	

Note: χ^2 = chi-square, GFI = Goodness of Fit Index, TLI = Tucker Lewis Index, CFI = Comparative Fit Index, RMSEA = Root Mean Square Error Approximation, INT = intention, B = behaviour.

Table 5. Hypothesis testing results and their status.

Paths	Hypothesis	Standardised Estimate (β)	Standard error	t-value	P-value (p) Significance
ATT→INT	H1	0.216	0.077	3.604	≤0.001***
SN→INT	H2	0.106	0.087	1.712	0.087
PBC→INT	H3	0.229	0.149	3.241	0.001**
INT→B	H4	0.677	0.093	13.407	≤0.001***
KN→ATT	H5	0.077	0.028	1.117	0.264
KN→INT	H6	-0.029	0.30	-0.520	0.603
PB→INT	H7	-0.051	0.074	-0.817	0.414
PSA→INT	H8	0.324	0.112	4.458	≤0.001***

Note: **significant effect at $p < 0.01$, ***significant effect at $p < 0.001$; ATT = attitudes, SN = subjective norms, PBC = perceived behavioural control (self-efficacy), INT = intention, KN = objective knowledge, PB = perceived barriers, PSA = perceived sensory attributes.
